# Environmental Fluoride Compromises Male Fertility: Differentially Modulated miR-34a-5p Targets REST to Regulate Autophagy in Testicular Somatic Cells

**DOI:** 10.34133/research.1113

**Published:** 2026-02-06

**Authors:** Ao Cheng, Yue Wu, Huifeng Luo, Xiaochao Song, Xiang Li, Bingchao Fan, Xinying Zhang, Shu Liu, Cuicui Zhuang, Yangfei Zhao, Jinming Wang, Chen Liang, Bin Liang, Jianhai Zhang

**Affiliations:** ^1^ College of Veterinary Medicine, Shanxi Agricultural University, Taigu, Shanxi 030801, China.; ^2^ College of Animal Science, Shanxi Agricultural University, Taigu, Shanxi 030801, China.; ^3^ Department of Cardiology, The Second Hospital of Shanxi Medical University, Taiyuan 030001, China.

## Abstract

The worldwide decline in male fertility represents a growing public health challenge, with fluoride exposure recognized as a key environmental factor exacerbating this decline. Fluoride hurts male reproduction, yet the specific mechanism remains unclear. Here, we demonstrate that fluoride reduced mouse sperm quality, destroyed the structure of testicular tissue, and caused severe damage to testicular somatic cells (Leydig and Sertoli cells). Meanwhile, the number of autophagosomes increased in Leydig cells and decreased in Sertoli cells. Network toxicology and functional analysis identified miR-34a-5p as the pivotal miRNA orchestrating fluoride-induced autophagic imbalance in testicular somatic cells. REST was identified as a novel miR-34a-5p target gene exhibiting pro-autophagic activity. Fluoride down-regulates miR-34a-5p and up-regulates REST in Leydig cells, whereas it exerts the opposite effects in Sertoli cells. The rescue experiment elucidated specific mechanisms: Fluoride down-regulates miR-34a-5p in Leydig cells, thereby derepressing REST to activate autophagy. Conversely, in Sertoli cells, fluoride up-regulates miR-34a-5p to suppress REST expression and inhibit autophagy. Collectively, the present study reveals an important mechanism underlying fluoride-induced male reproductive toxicity and provides a potential therapeutic target.

## Introduction

The increasing prevalence of environmental pollution and shifting lifestyles have brought male reproductive health issues into sharp focus as a global public health concern. Infertility, affecting an estimated 17.5% of reproductive-age couples worldwide [World Health Organization (WHO) data], exhibits a substantial male factor component, implicated in nearly half of all occurrences [1]. Furthermore, a significant decline in global male fertility has been documented throughout the 20th and 21st centuries [2]. Fluoride, a pervasive environmental pollutant, occurs naturally and is also released through industrial and anthropogenic activities, including aluminum smelting, phosphate fertilizer production, fluorinated chemical manufacturing, and water fluoridation [3,4]. Drinking water constitutes the primary exposure pathway for fluoride in humans, with subsequent bioaccumulation occurring within the body [5]. Chronic fluoride exposure disrupts spermatogenesis and damages testicular structure and reproductive hormone homeostasis, thereby posing a significant threat to male fertility [6]. The observed global decline in male fertility is also linked to exposure to fluoride [7]. Consequently, the reproductive toxicity of fluoride in males warrants significant attention and further investigation.

Testicular somatic cells (Leydig and Sertoli cells) play crucial roles in the maintenance of testicular structure, androgen biosynthesis, and spermatogenesis [8]. Therefore, testicular somatic cells have received extensive attention in the study of male reproductive toxicity of fluoride. Recent research has found that fluoride exposure causes the death of testicular somatic cells [9,10], disrupts testosterone biosynthesis in Leydig cells [11], and impairs Sertoli cells’ immune-privileged function [12]. Autophagy, a cellular process by which lysosomes break down and recycle damaged organelles and macromolecules [13], performs a critical regulator as far as toxicology and reproduction [14,15]. The regulation of autophagy exerts a dual influence on cell death [16]. Moderate autophagy protects cells from toxicants and promotes cell survival [17]. Impaired or excessive autophagy contributes to toxicant-induced cell death [18,19]. Furthermore, autophagy is imperative to maintain the functionality of testicular somatic cells [20]. Disruption of autophagy in Leydig cells inhibits cholesterol uptake and reduces testosterone production [21]. In Sertoli cells, autophagic suppression destabilizes the cell’s cytoskeleton, leading to the breakdown of ectoplasmic specialization assembly, ultimately contributing to male infertility [22,23]. Emerging evidence indicates that autophagy is a key executor of damage to testicular somatic cell induced by fluoride [24,25]. However, the effects of fluoride on autophagy were different between the 2 cell types. Fluoride increases autophagosome formation of in Leydig cells, by inhibiting the mammalian target of rapamycin (mTOR) activity [24]. In contrast, fluoride exposure down-regulated the LC3B II/LC3B I ratio in Sertoli cells, indicating an inhibition of autophagy [25]. Therefore, deciphering the divergent autophagic responses to fluoride exposure—enhancement in Leydig cells versus suppression in Sertoli cells—at the molecular level is imperative for elucidating fluoride’s male reproductive toxicity mechanisms.

miRNAs are recognized as critical regulators in male reproduction and toxicology due to their roles in epigenetic regulation, tissue-specific expression, and transport via blood circulation [26,27]. Additionally, miRNAs are important modulators of autophagy [28]. It is notable that miR-34a-5p, which is expressed at a high abundance in the testes, plays a vital role in preserving the optimal conditions for sperm motility and spermatogenesis [29]. It is also implicated in oxidative stress and mitochondrial apoptosis pathways, contributing to testicular injury [30]. Further studies have revealed that miR-34a-5p activates testicular apoptotic cell death (TACD), leading to male infertility [31]. Moreover, substantial evidence indicates that miR-34a-5p functions as a significant controller of autophagy [32]. It influences autophagic activity in various cell types, including nerve cells [33], hepatocytes [34], cardiomyocytes [35], chondrocytes [36], and tumor cells [37]. Recent research has identified a correlation between the male reproductive toxicity of fluoride and miR-34a-5p [38]. At present, it remains unclear whether miR-34a-5p participates in the regulation of autophagy in testicular somatic cells, and direct evidence delineating its involvement in fluoride-induced male reproductive toxicity is still lacking. Therefore, it is urgent to clarify the regulatory role of miR-34a-5p on testicular somatic cell autophagy and the mechanism of miR-34a-5p-mediated fluoride-induced autophagy dysregulation.

This study aimed to investigate the contribution of miRNAs in fluoride-induced autophagy in testicular somatic cells. Network toxicology analysis identified miR-34a-5p as a critical mediator of fluoride-regulated autophagy. REST is a novel target of miR-34a-5p, through which autophagy is modulated in testicular somatic cells. Subsequently, loss-of-function assays conducted in vitro demonstrated that fluoride suppresses miR-34a-5p to increase REST and thus promote autophagy in Leydig cells while elevating miR-34a-5p to down-regulate REST and inhibit autophagy in Sertoli cells. This study will advance the current comprehension of the mechanisms underpinning fluoride-induced male reproductive toxicity and offer potential intervention targets.

## Results

### Fluoride impairs the male reproductive ability by differentially regulating autophagy in testicular somatic cells

**Fig. 1.**
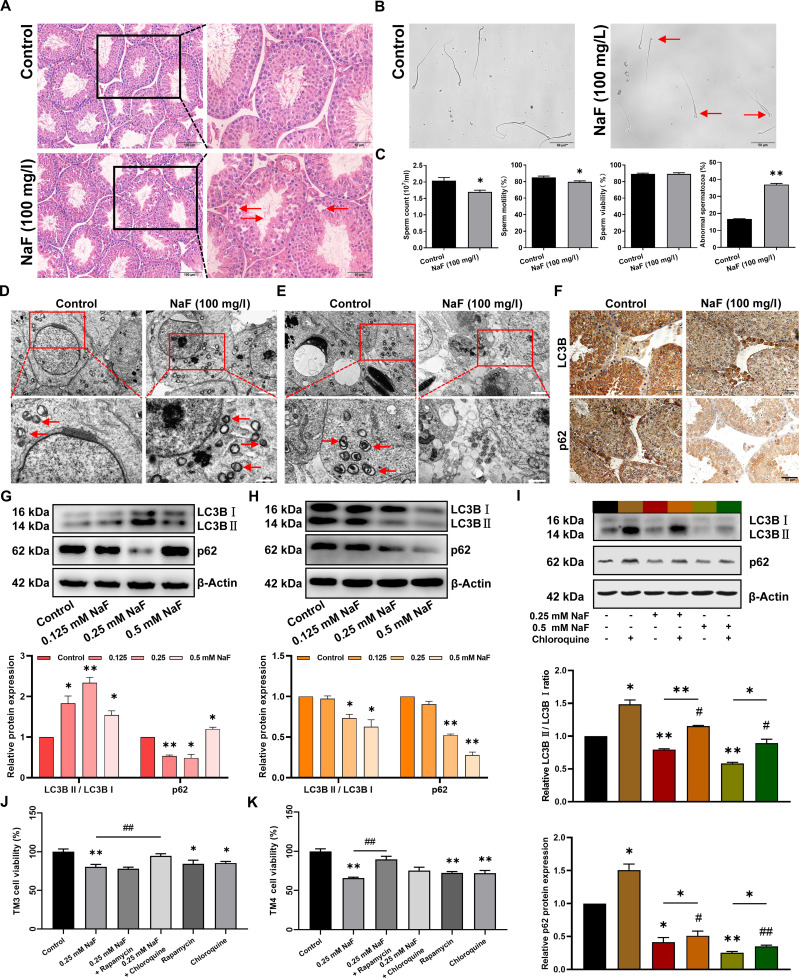
Fluoride impairs male reproductive capacity by differentially regulating autophagy of testicular somatic cells in mice. (A to F) Six-week-old male mice freely drank distilled water with or without 100 mg/l NaF for 18 weeks. (A) Representative images of H&E staining on testis. Scale bars, 100 μm (left) and 50 μm (right). (B) Representative microscopic views of sperm morphology. Scale bars, 50 μm. (C) Sperm quality. (D and E) Representative transmission electron microscopy images of autophagosomes in (D) Leydig cells and (E) Sertoli cells [scale bars, 2 μm (bottom) and 1 μm (top)], with red arrows indicating autophagosomes. (F) Immunohistochemistry was used to detect the protein expression of LC3B and p62 in Leydig cells and Sertoli cells. (G and H) Testicular somatic cells were treated with various concentrations of NaF for 24 h. The relative expression of LC3B and p62 proteins in (G) TM3 cells, (H) TM4 cells, and (I) TM4 cells cotreated with 20 μM chloroquine. Cell viability of (J) TM3 and (K) TM4 cells that were treated with 0.25 mM NaF and 20 μM chloroquine for 24 h or pretreated with 20 nM rapamycin for 1 h. All values in the figure are means ± SEM. *n* ≥ 3. *P < 0.05, **P < 0.01 versus control; ^#^*P* < 0.05, ^##^*P* < 0.01 versus chloroquine.

To investigate the effects of fluoride on autophagy in testicular somatic cells (Leydig and Sertoli cells), a male mouse model exposed to fluoride was established. Hematoxylin and eosin (H&E) staining (Fig. 1A) revealed abundant and well-organized spermatogenic cells in the testes of control mice. Contrary to this, the fluoride-exposed mice exhibited a reduced number of germinal layers of seminiferous tubules, disorganized testicular cells, and decreased luminal sperm density. Furthermore, fluoride exposure significantly reduced sperm quality, and sperm malformation was primarily characterized by head deformities, double head, or double tail (Fig. 1B and C). These findings suggest that fluoride adversely impacts male reproductive capacity. Subsequently, in fluoride-exposed testes, transmission electron microscopy (TEM) analysis revealed an increase in autophagosome number in Leydig cells (Fig. 1D) and a decline in Sertoli cells (Fig. 1E). Immunohistochemical analysis displayed that LC3B was increased in Leydig cells and decreased in Sertoli cells, and p62 staining was weak in both Leydig and Sertoli cells (Fig. 1F). An in vitro model of testicular somatic cells exposed to fluoride was subsequently established. Western blot (WB) analysis demonstrated that in TM3 Leydig cells, the LC3B II/LC3B I ratio exhibited a marked elevation in all fluoride groups (Fig. 1G), and the p62 protein amount was significantly reduced in the 0.125 and 0.25 mM NaF groups but notably increased in the 0.5 mM NaF group. In TM4 Sertoli cells, both the p62 protein amount and the LC3B II/LC3B I ratio were considerably lower in the 0.25 and 0.5 mM NaF groups than in the control group (Fig. 1H). We then explored why the decrease in autophagosome number was accompanied by a decrease in p62 in Sertoli cells. After treatment with 20 μM chloroquine (an inhibitor of autophagy–lysosome fusion), the ratio of LC3B II/LC3B I and the p62 protein amount both significantly increased, indicating that the autophagic flux was effectively inhibited. After cotreatment with 0.25 or 0.5 mM NaF and chloroquine, the ratio of LC3B II/LC3B I and the p62 protein amount were significantly higher than those in the corresponding NaF-only groups, but significantly lower than those in the chloroquine-only groups (Fig. 1I). These results indicated that fluoride exposure increases autophagosome formation in Leydig cells but disrupts autophagic flux at higher concentrations, primarily by blocking the autolysosomal degradation pathway. Conversely, fluoride inhibits autophagy in Sertoli cells in a dose-dependent way, primarily by suppressing autophagosome formation and not hindering autophagosome degradation. Subsequently, the role of fluoride-induced autophagy in the testicular somatic cytotoxicity was explored. After treatment with 0.25 mM NaF, chloroquine, or rapamycin, the viability of testicular somatic cells significantly decreased. In TM3 cells, the cotreatment with chloroquine significantly alleviated the cell viability decline induced by fluoride exposure, while in TM4 cells, the pretreatment with rapamycin significantly mitigated the cell viability decline caused by fluoride exposure (Fig. 1J and K). This indicates that excessive or inhibited autophagy is detrimental to testicular somatic cells’ survival. Specifically, fluoride-induced excessive autophagy occurs in Leydig cells, while inhibited autophagy is observed in Sertoli cells. In summary, fluoride impairs the male reproductive capacity by differentially regulating autophagy in testicular somatic cells.

### miR-34a-5p was identified as the key miRNA in the differential regulation of autophagy in testicular somatic cells by fluoride

**Fig. 2.**
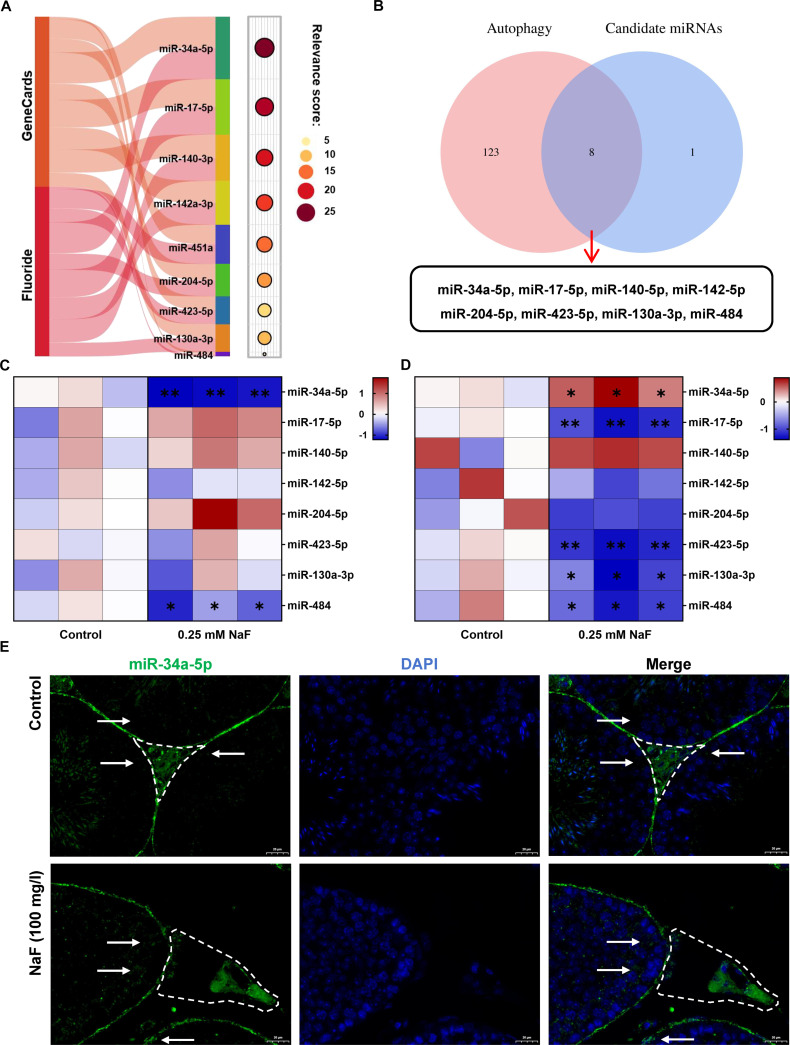
miR-34a-5p was selected as the key miRNA in the differential regulation of autophagy in testicular somatic cells by fluoride. (A) Network toxicological analysis results of miRNAs related to fluoride-induced male reproductive injury. (B) HAMDB database analysis results of autophagy-related candidate miRNAs. (C and D) qRT-PCR revealed autophagy-related candidate miRNA expression changes in (C) TM3 and (D) TM4 cells after fluoride treatment. (E) FISH of miR-34a-5p in the testis of control and fluoride-treated mice (dashed areas indicate Leydig cells, and white arrows indicate Sertoli cells). All values in the figure are means ± SEM. *n* = 3. *P < 0.05, **P < 0.01.

miRNAs are widely implicated in the modulation of autophagy [28]. To explore the key miRNAs involved in the differential regulation of autophagy following fluoride exposure—promotion in Leydig cells and inhibition in Sertoli cells, we conducted a bioinformatics analysis. As illustrated in Fig. 2A, 222 miRNAs linked to fluoride-induced male reproductive damage were retrieved from the National Center for Biotechnology Information (NCBI) database. Additionally, the GeneCards database identified 417 miRNAs associated with male reproductive toxicity. Intersection analysis revealed 9 candidate miRNAs, among which miR-34a-5p exhibited the highest relevance score of 26.99 (Table S3). Further analysis using the HAMDB database identified miR-34a-5p, miR-17-5p, miR-204-5p, miR-423-5p, miR-140-3p, miR-142a-3p, miR-130a-3p, and miR-484 as regulators of autophagy (Fig. 2B and Table S4). Given that 0.25 mM NaF exposure promotes autophagy in Leydig cells while inhibiting autophagy in Sertoli cells, subsequent experiments were conducted at this concentration. The impact of fluoride on the autophagy-related candidate miRNA expression was assessed using quantitative reverse transcription polymerase chain reaction (qRT-PCR). The results exhibited that in TM3 Leydig cells, the miR-484 and miR-34a-5p expression was significantly down-regulated following fluoride exposure (Fig. 2C). In TM4 Sertoli cells, miR-34a-5p expression was substantially increased, while miR-130a-3p, miR-17-5p, miR-423-5p, and miR-484 expression was significantly down-regulated (Fig. 2D). Notably, miR-34a-5p exhibited opposite expression responses in fluoride-exposed Leydig cells and Sertoli cells. Further detection of miR-34a-5p expression in the testis by fluorescence in situ hybridization (FISH) revealed a decrease in fluorescence intensity in Leydig cells and an increase in Sertoli cells after fluoride exposure (Fig. 2E). These findings suggest that fluoride increases miR-34a-5p expression in Sertoli cells and decreases its expression in Leydig cells. In addition, miR-34a-5p is a key miRNA mediating the promotion of autophagy in Leydig cells and the inhibition of autophagy in Sertoli cells induced by fluoride.

### Fluoride differentially regulates miR-34a-5p to modulate autophagy in testicular somatic cells

**Fig. 3.**
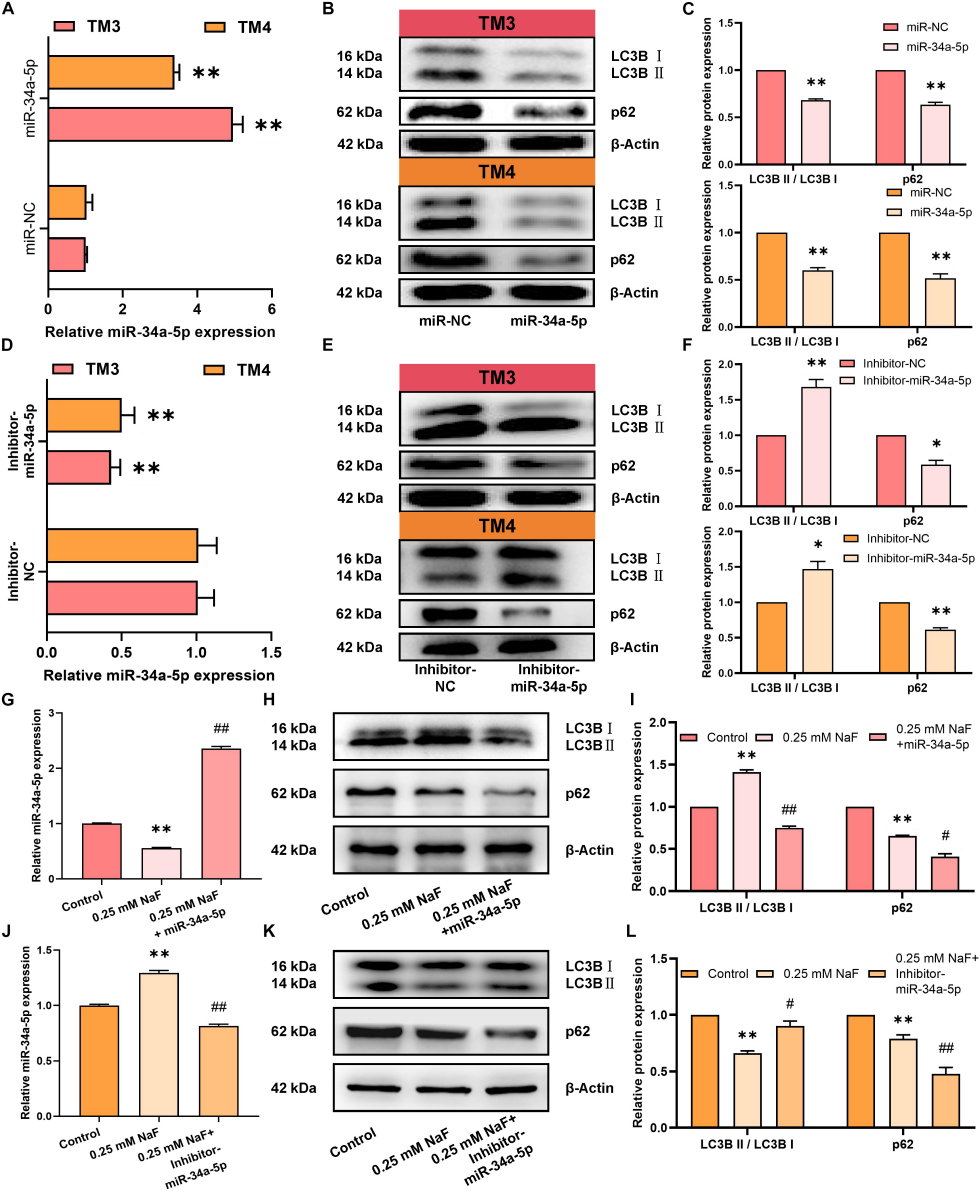
Fluoride differentially regulates miR-34a-5p to modulate autophagy in testicular somatic cells. (A to F) Testicular somatic cells were treated with lentiviruses expressing miR-34a-5p/miR-NC and Inhibitor-miR-34a-5p/Inhibitor-NC. (A and D) Relative expression of miR-34a-5p. (B, C, E, and F) Relative expression of LC3B and p62 proteins. (G to I) miR-34a-5p overexpression rescued fluoride-treated TM3 cells. (G) Relative expression of miR-34a-5p. (H and I) Relative expression of LC3B and p62 proteins. (J to L) miR-34a-5p knockdown rescued fluoride-treated TM4 cells. (J) Relative expression of miR-34a-5p. (K and L) Relative expression of LC3B and p62 proteins. All values in the figure are means ± SEM. *n* = 3. *P < 0.05, **P < 0.01 versus control, miR-NC, or Inhibitor-NC; ^#^*P* < 0.05, ^##^*P* < 0.01 versus 0.25 mM NaF.

To investigate whether fluoride differentially regulates autophagy in testicular somatic cells through miR-34a-5p, first, the regulatory effect of miR-34a-5p on autophagy in testicular somatic cells was explored. The overexpression and knockdown of miR-34a-5p were mediated by lentivirus in testicular somatic cells. In TM3 and TM4 cells, qRT-PCR confirmed that miR-34a-5p expression was significantly higher in the miR-34a-5p group compared to the miR-NC group. At the same time, it was markedly lower in the Inhibitor-miR-34a-5p group compared to the Inhibitor-NC group (Fig. 3A and D). WB analysis demonstrated that the p62 protein amount and LC3B II/LC3B I ratio in the miR-34a-5p group were lower than those in the miR-NC group (Fig. 3B and C). Compared to the Inhibitor-NC group, the LC3B II/LC3B I ratio was increased, and the amount of p62 protein was decreased in the Inhibitor-miR-34a-5p group (Fig. 3E and F). The above results suggest that miR-34a-5p inhibits autophagy in testicular somatic cells.

To rescue fluoride-induced effects, miR-34a-5p was overexpressed in fluoride-exposed TM3 Leydig cells. qRT-PCR results showed that miR-34a-5p overexpression reversed the fluoride-induced reduction in miR-34a-5p expression (Fig. 3G). WB analysis further revealed that fluoride treatment significantly decreased the p62 protein amount and increased the LC3B II/LC3B I ratio compared to the control group. In contrast, the rescue group exhibited a significant reduction in the p62 protein amount and LC3B II/LC3B I ratio compared to the fluoride-treated group (Fig. 3H and I). miR-34a-5p overexpression reverses fluoride-induced autophagy in TM3 Leydig cells. miR-34a-5p was knocked down in fluoride-exposed TM4 Sertoli cells. qRT-PCR results revealed that miR-34a-5p knockdown reversed the fluoride-induced up-regulation of miR-34a-5p expression (Fig. 3J). WB analysis further confirmed that compared to the control group, the fluoride treatment group exhibited a reduced p62 protein amount and decreased LC3B II/LC3B I ratio. In contrast, the rescue group displayed a decreased p62 protein amount and increased LC3B II/LC3B I ratio compared to the fluoride-treated group (Fig. 3K and L). Knockdown of miR-34a-5p reversed the inhibition of autophagy induced by fluoride in TM4 Sertoli cells. Collectively, our results indicate that fluoride promotes autophagy in Leydig cells by down-regulating miR-34a-5p and inhibits autophagy in Sertoli cells by up-regulating it.

### miR-34a-5p targets REST in testicular somatic cells exposed to fluoride

**Fig. 4.**
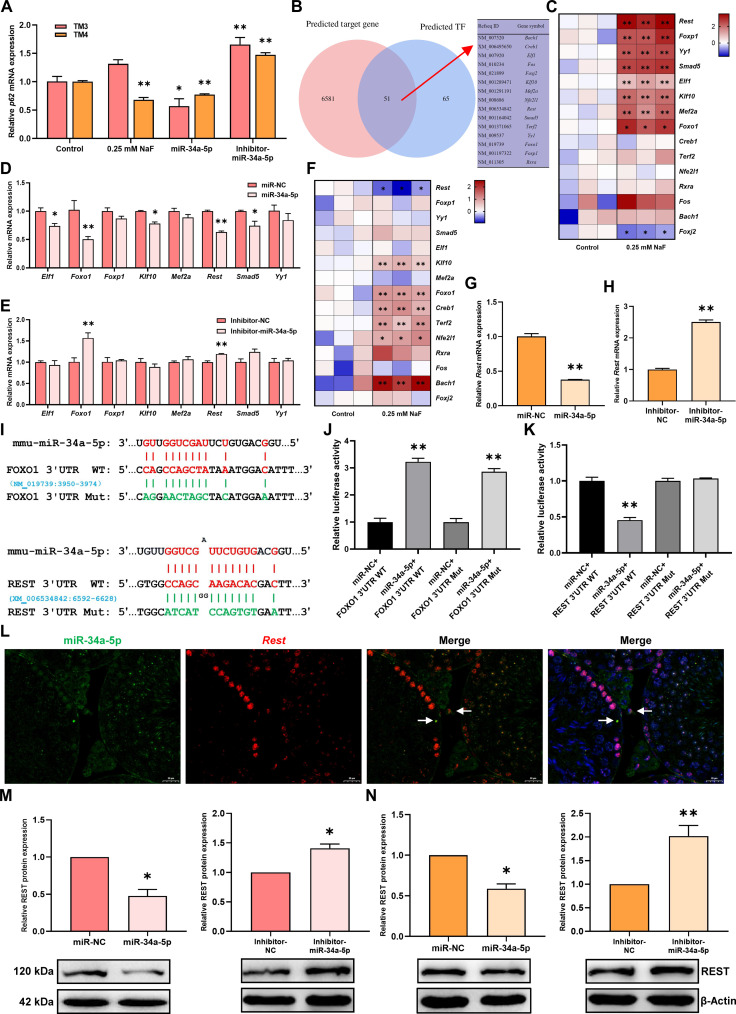
miR-34a-5p targets REST in testicular somatic cells exposed to fluoride. (A) Expression changes of *p62* mRNA in TM3 and TM4 cells after fluoride treatment and overexpression or knockdown of miR-34a-5p. (B) Prediction results of miR-34a-5p target genes by miRWalk combined with ChEA3 database. (C and F) Differential expression of the top 15 predicted target genes after fluoride treatment in (C) TM3 and (F) TM4 cells. (D, E, G, and H) Relative mRNA expression of *Elf1*, *Foxo1*, *Foxp1*, *Klf10*, *Mef2a*, *Rest*, *Smad5*, and *Yy1*. (I) Schematic showing the targeted binding sites of miR-34a-5p to FOXO1 and REST 3′ UTR. (J and K) The histogram shows the relative fluorescence intensity of each group. (L) FISH of miR-34a-5p and *Rest* mRNA in testicular tissue from control mice (white arrow indicates the positive area within the Sertoli cells or Leydig cells). (M and N) Differential expression of REST protein in testicular somatic cells after overexpression or knockdown of miR-34a-5p. All values in the figure are means ± SEM. *n* = 3. *P < 0.05, **P < 0.01.

In this study, the observed decrease in autophagy was accompanied by a reduction in p62 protein amount, which may be attributed to the change in *p62* mRNA expression. To test this hypothesis, *p62* mRNA expression was examined, and qRT-PCR results showed that *p62* mRNA expression was increased in fluoride-exposed TM3 Leydig cells but decreased in fluoride-exposed TM4 Sertoli cells. Furthermore, *p62* mRNA expression in testicular somatic cells was significantly reduced following miR-34a-5p overexpression and significantly elevated after miR-34a-5p knockdown (Fig. 4A). This implies that the reduction in p62 protein amount, concomitant with decreased autophagy, is mediated by changes in *p62* mRNA expression, and the target gene of miR-34a-5p was related to p62.

To identify the target genes of miR-34a-5p, the miRWalk database predicted 6,632 potential target genes, excluding p62. We hypothesized that the target gene might be a transcription factor regulating p62. One hundred sixteen transcription factors of p62 were found using the ChEA3 database. By integrating data from both databases, we narrowed down the list to 51 potential target genes (Fig. 4B and Table S5). From these, the top 15 candidates were selected for further analysis. Heatmap analysis exhibited that the mRNA expression of *Rest*, *Foxp1*, *Yy1*, *Smad5*, *Elf1*, *Klf10*, *Mef2a*, and *Foxo1* were significantly up-regulated in TM3 Leydig cells following fluoride exposure, while *Foxj2* expression was significantly down-regulated (Fig. 4C). Among the up-regulated genes, only *Foxo1* and *Rest* exhibited significant reductions in TM3 Leydig cells overexpressing miR-34a-5p and exhibited significantly increased in cells with miR-34a-5p knockdown (Fig. 4D and E). In TM4 Sertoli cells, heatmap analysis showed that *Rest* mRNA expression was significantly decreased after fluoride exposure, while the expressions of *Klf10*, *Foxo1*, *Creb1*, *Terf2*, *Nfe2l1,* and *Bach1* were significantly increased (Fig. 4F). Further analysis of down-regulated genes confirmed that *Rest* mRNA expression was significantly reduced in TM4 Sertoli cells overexpressing miR-34a-5p and significantly elevated in TM4 Sertoli cells with miR-34a-5p knockdown (Fig. 4G and H). To validate the targeting relationship between miR-34a-5p and FOXO1 or REST, the targeting sites of miR-34a-5p in REST and FOXO1 3′ untranslated region (UTR) were analyzed by the miRWalk database, and mutant targeting sites were designed (Fig. 4I). Dual-luciferase reporter assay demonstrated no direct targeting relationship between miR-34a-5p and FOXO1 but confirmed a direct interaction between miR-34a-5p and REST (Fig. 4J and K). FISH assay of testicular tissue showed that miR-34a-5p and *Rest* were colocalized in Leydig and Sertoli cells (Fig. 4L). Additionally, WB analysis revealed that REST protein expression was significantly decreased in both TM3 Leydig and TM4 Sertoli cells following miR-34a-5p overexpression and significantly increased after miR-34a-5p knockdown (Fig. 4M and N). Taken together, REST is a newly identified target gene of miR-34a-5p that is translationally repressed by binding to its 3′ UTR.

### miR-34a-5p targets REST to regulate autophagy in testicular somatic cells

**Fig. 5.**
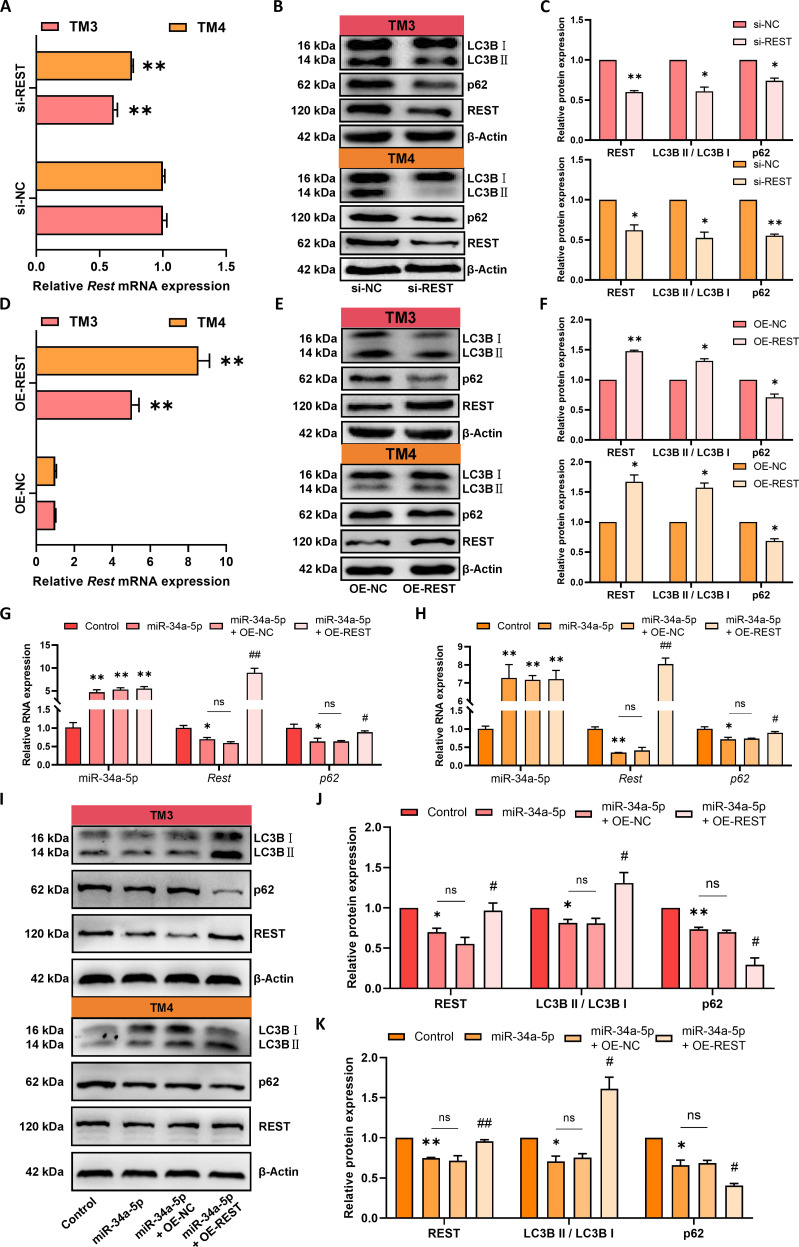
miR-34a-5p targets REST to regulate autophagy in testicular somatic cells. (A to F) TM3 and TM4 cells were treated with lentivirus expressing OE-REST, si-REST, OE-NC, or si-NC. (A and D) Relative *Rest* mRNA expression. (B, C, E, and F) Relative LC3B, p62, and REST protein expression. (G to K) REST overexpression rescued TM3 and TM4 cells overexpressing miR-34a-5p. (G and H) Relative miR-34a-5p, *Rest*, and *p62* mRNA expression. (I to K) Relative REST, LC3B, and p62 protein levels. All values in the figure are means ± SEM. *n* = 3. ns indicates not significant; *P < 0.05, **P < 0.01 versus control; ^#^*P* < 0.05, ^##^*P* < 0.01 versus miR-34a-5p.

To further investigate whether miR-34a-5p targets REST to regulate autophagy in testicular somatic cells, first, the regulatory effect of REST on autophagy in testicular somatic cells was explored. REST was either knocked down or overexpressed in TM3 Leydig and TM4 Sertoli cells. qRT-PCR and WB analyses confirmed that REST expression was significantly down-regulated in the si-REST group compared to the si-NC group (Fig. 5A and B), and the expression of REST was significantly up-regulated in the OE-REST group compared to the OE-NC group (Fig. 5D and E). Furthermore, REST knockdown significantly reduced the p62 protein amount and LC3B II/LC3B I ratio (Fig. 5B and C), whereas REST overexpression decreased the p62 protein amount and increased the LC3B II/LC3B I ratio (Fig. 5E and F). These findings indicate that REST promoted autophagy in testicular somatic cells.

REST overexpression was used to rescue miR-34a-5p overexpression in testicular somatic cells. qRT-PCR analysis confirmed that the miR-34a-5p expression in the miR-34a-5p group, miR-34a-5p + OE-NC group, and miR-34a-5p + OE-REST group was significantly higher than that in the control group (Fig. 5G and H). qRT-PCR and WB results revealed that REST overexpression reversed the miR-34a-5p-induced reduction in REST expression (Fig. 5G to K). Furthermore, REST overexpression rescued the miR-34a-5p-induced reduction in autophagy in testicular somatic cells. qRT-PCR analysis showed that compared to the control group, the *p62* mRNA expression in the miR-34a-5p group was decreased, and the expression of *p62* mRNA in the rescue group was increased compared to the miR-34a-5p group. No significant changes were observed in any of the measured parameters following negative rescue. These findings reveal that miR-34a-5p-targeted REST plays a function in regulating autophagy and *p62* mRNA expression in testicular somatic cells.

### Fluoride differentially regulates REST to modulate autophagy in testicular somatic cells

**Fig. 6.**
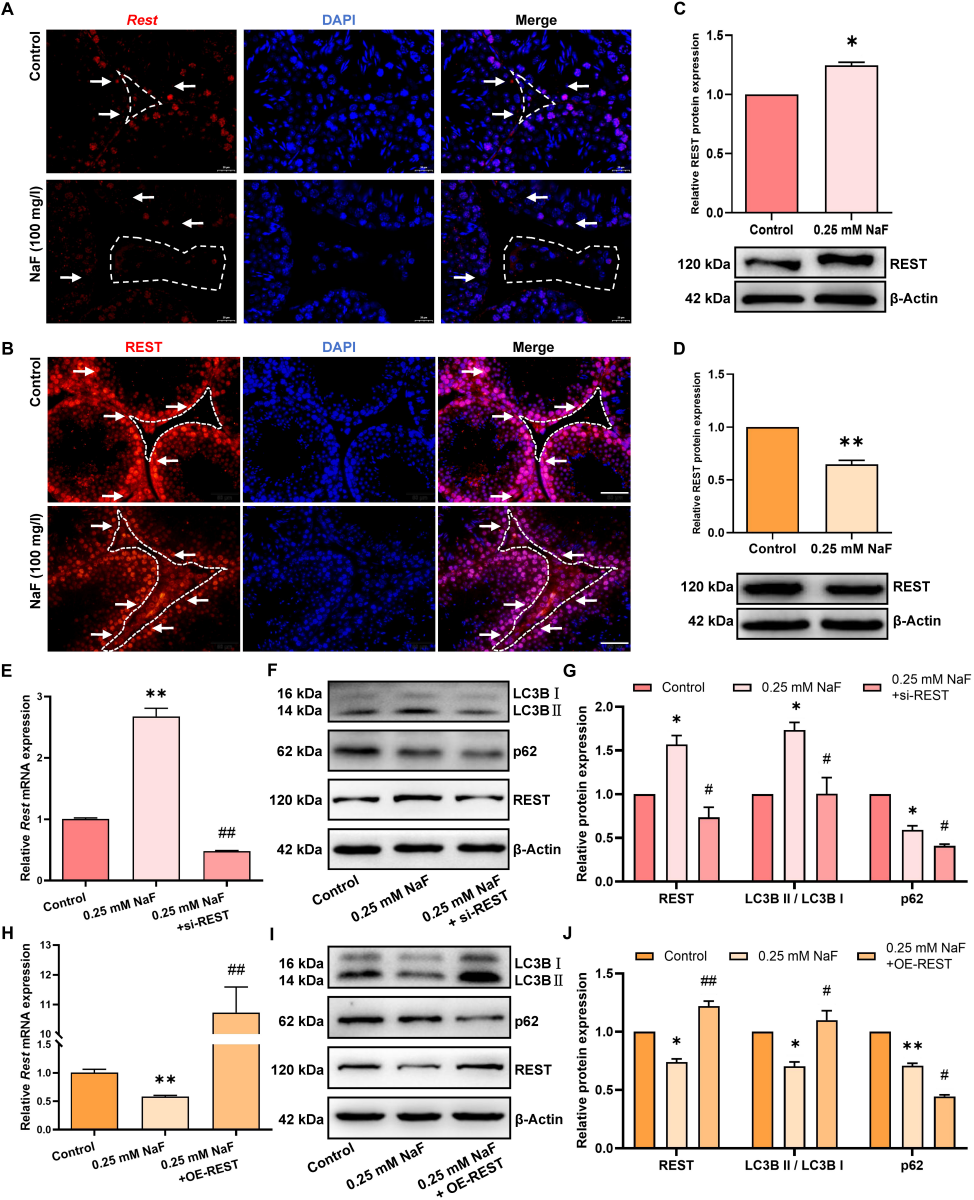
Fluoride differentially regulates REST to modulate autophagy in testicular somatic cells. (A) Representative image of *Rest* mRNA FISH in testes from control and fluoride-treated mice. (B) Representative immunofluorescence staining of REST proteins in testes from control and fluoride-treated mice. Dashed areas indicate Leydig cells, and white arrows indicate Sertoli cells. (C and D) Relative REST protein expression in testicular somatic cells treated with fluoride. (E to G) REST knockdown rescued fluoride-treated TM3 cells. (E) Relative expression of *Rest* mRNA. (F and G) Relative expression of REST, p62, and LC3B proteins. (H to J) REST overexpression rescued fluoride-treated TM4 cells. (H) Relative expression of *Rest* mRNA. (I and J) Relative expression of REST, p62, and LC3B proteins. All values in the figure are means ± SEM. *n* = 3. *P < 0.05, **P < 0.01 versus control, si-NC, or OE-NC; ^#^*P* < 0.05, ^##^*P* < 0.01 versus 0.25 mM NaF.

To test whether fluoride regulates autophagy in testicular somatic cells through REST, fluoride-induced changes in REST expression were first examined. FISH and immunofluorescence analysis showed that fluoride treatment up-regulated REST mRNA and protein levels in Leydig cells while down-regulating REST expression in Sertoli cells (Fig. 6A and B). WB results further demonstrated these specific effects, showing increased REST expression in fluoride-treated TM3 Leydig cells (Fig. 6C) but decreased expression in TM4 Sertoli cells (Fig. 6D). These results demonstrate that fluoride increased REST expression in Leydig cells and reduced REST expression in Sertoli cells.

To rescue the effects of fluoride, REST was knocked down in fluoride-exposed TM3 Leydig cells (Fig. 6E to G). qRT-PCR and WB results revealed that REST knockdown reversed the fluoride-induced increase in REST expression. In addition, the p62 protein amount was decreased, and the LC3B II/LC3B I ratio was increased in the fluoride-treated group. In contrast, the rescue group displayed a significant reduction in the p62 protein amount and LC3B II/LC3B I ratio compared to the fluoride-treated group, indicating that REST knockdown reversed fluoride-induced autophagy in TM3 Leydig cells. REST was overexpressed in fluoride-treated TM4 Sertoli cells (Fig. 6H to J), qRT-PCR and WB results revealed that REST overexpression reversed the fluoride-induced down-regulation of REST expression. Additionally, the fluoride-treated group exhibited a significantly reduced p62 protein amount and a lower LC3B II/LC3B I ratio. In contrast, the rescue group displayed a decreased p62 protein amount and increased LC3B II/LC3B I ratio compared to the fluoride-treated group, indicating that REST overexpression reversed fluoride-induced inhibition of autophagy in TM4 Sertoli cells. These results suggest that fluoride promotes autophagy in Leydig cells by up-regulating REST expression and inhibits autophagy in Sertoli cells by down-regulating REST expression.

## Discussion

Fluoride, an omnipresent environmental contaminant, is known for its toxic effects on male reproduction [6]. Fluoride-induced male reproductive damage is implicated in the death and dysfunction of testicular somatic cells [9–12]. Autophagy plays a major role in maintaining the homeostasis and function of testicular somatic cells [39], and participates in the damage to these cells induced by fluoride exposure [24,25]. miRNAs, a regulator of autophagy [32], have not yet been studied for their potential role in fluoride-induced autophagy disruption in testicular somatic cells. Here, the present study reveals that miR-34a-5p targets the RE1 silencing transcription factor (REST) to mediate fluoride’s dual effects on autophagy in testicular somatic cells: In Leydig cells, down-regulation of miR-34a-5p promotes autophagy, whereas in Sertoli cells, up-regulation of miR-34a-5p inhibits autophagy. These findings provide a novel understanding of the mechanisms underlying fluoride-induced male reproductive damage and identify potential targets for therapeutic intervention.

Studies show that the majority of people exposed to fluoride globally are from Asia and Africa [4]. Fluoride concentrations in groundwater were found to exceed 1.5 mg/l in most parts of Asian countries, with the highest level of fluoride ions found in Afghanistan at 79.2 mg/l [40]. In Africa, the fluoride pollution range in East Africa is 0.01 to 588 mg/l, West Africa is 0.1 to 13.29 mg/l, North Africa is 0.1 to 10.5 mg/l, South Africa is 0.04 to 65.9 mg/l, and Central Africa is 0.01 to 1.9 mg/l. Of these, 20% to 35% in the east are at threat of fluoride levels exceeding WHO limits [41]. In addition, the Global Burden of Diseases, Injuries, and Risk Factors Study predicted that the life expectancy of global men will increase from 71.1 years in 2022 to 76.0 years by 2050 [42]. The study also showed that the average childbearing age of men today is 30.7 years old [43]. In animal model studies, the average lifespan of mice is about 104 weeks [44]. In addition, rodents are more tolerant to fluoride, about 10 times more so than humans [45]. Comparable serum fluoride levels between mice and humans are achieved by administering 50 mg/l in drinking water to mice, mirroring a human exposure concentration of 2 to 3 mg/l [46]. Therefore, in this study, mice were exposed to 100 mg/l NaF (45.22 mg F/l) in drinking water freely for 18 weeks to simulate man’s exposure to a high fluoride environment from 18 years of sexual maturity to the average reproductive age. This study further emphasizes that the harm of long-term high fluoride exposure to male reproductive capacity is a serious concern.

Autophagy is crucial for testicular somatic cell homeostasis and function [47–49]. Fluoride induces testicular somatic cell injury by disrupting the autophagy process. Current studies indicate that fluoride disrupts autophagy in testicular somatic cells. In Leydig cells, fluoride inhibits mTOR phosphorylation and increases autophagosome formation [24]. In Sertoli cells, fluoride reduces the LC3B II/LC3B I ratio, impairing autophagy [25]. This study further reveals that fluoride promotes the formation of autophagosomes in Leydig cells, but high fluoride concentrations disrupt autophagy via blockage of the autophagy–lysosomal degradation process. In Sertoli cells, fluoride inhibits autophagy in a dose-dependent path, primarily by suppressing autophagosome formation.

During autophagy, the selective autophagy receptor sequestosome 1 (p62) is incorporated into autophagosomes and subsequently degraded by autolysosomes [50]. Autophagy inhibition typically results in p62 protein accumulation [51]. Intriguingly, fluoride reduced autophagosome numbers in Sertoli cells while simultaneously decreasing p62 protein expression. It was noted that in rectal cancer cells, miR-34a inhibited autophagy by targeting FOXM1 to indirectly inhibit p62 gene expression [37]. Furthermore, a similar phenomenon was observed in Sertoli cells exposed to phosphatidylserine-coated polystyrene beads, which was shown to arise from suppressed autophagosome membrane formation and activation of autophagic proteolysis [52]. Thus, the decreased p62 protein amount in fluoride-exposed Sertoli cells may be due to decreased *p62* mRNA expression and also specifically reflects enhanced autophagic proteolytic activity.

Autophagy is intricately regulated by miRNAs, with miR-34a-5p having emerged as a key regulatory factor of this process. In mouse liver, up-regulation of miR-34a-5p induced by BDE-47 impairs mitophagy, leading to mitochondrial dysfunction and liver damage [53]. miR-34a-5p overexpression in aged mouse brains suppresses mitophagy by down-regulating PINK1 [54]. In osteoarthritis (OA) cells, miR-34a-5p promotes autophagy by targeting SYVN1 [36]. Han et al. [55] found that miR-34a-5p is significantly elevated in atretic follicles, where it targets lymphoid enhancer binding factor 1 to activate the Hippo–TAP pathway and drive autophagy in chick follicle granulosa cells. These studies demonstrate that miR-34a-5p exhibits divergent regulatory effects on autophagy across tissues and cell types. However, its role in testicular somatic cell autophagy remains unexplored. This work demonstrates that miR-34a-5p suppresses autophagy in testicular somatic cells. Previous studies have shown that fluoride down-regulates miR-296-5p to activate the AMPK/ULK1 pathway and promote autophagy in LS8 ameloblasts [56]. In ovarian granulosa cells, fluoride elevates miR-378d to induce female reproductive damage by modulating autophagy, apoptosis, and estradiol secretion [57]. Notably, this study demonstrate that fluoride down-regulates miR-34a-5p to enhance autophagy in Leydig cells while up-regulating it to inhibit autophagy in Sertoli cells.

This study identified REST as a novel target molecule for miR-34a-5p. REST, also termed neuron-limiting silencing factor (NRSF), is a transcription factor with a zinc finger structure [58]. It exerts its biological function by binding to the conserved 23–base pair motif RE1 in specific genes [59]. Initially, REST was found to be expressed and functionally significant in the nervous system, cardiovascular system, skin, pancreas, and eyes [60]. Kimura et al. [61] successfully visualize widespread REST protein expression in testicular tissue using tag knock-in mice. Yoshizaki et al. [62] discovered that REST binding motifs were enriched in the hypomethylated region of sperm DNA in aged mice, suggesting a role for REST in testicular aging. Additionally, REST has been reported to regulate autophagy in neuronal cells [63]. However, whether REST regulates autophagy in testicular somatic cells or mediates fluoride-induced autophagy dysregulation has not been investigated. Our findings provided evidence that REST promotes autophagy in testicular somatic cells. Specifically, fluoride up-regulates REST expression to enhance autophagy in Leydig cells but down-regulates REST to suppress autophagy in Sertoli cells. Current studies indicate that REST regulates autophagy by mTOR phosphorylation [64,65]. Prior research has identified that REST acts as a transcription factor to activate JAM-B expression in Sertoli cells [66]. miRNA-mediated autophagy regulation often involves transcriptional cascades. For instance, in HK-2 kidney tubular epithelial cells, miR-155 targets SIRT1, leading to down-regulation of autophagy-related genes, which include *LC3B*, *ATG7*, and *ATG5* [67]. Here, miR-34a-5p targets REST to modulate autophagy and *p62* mRNA transcription in testicular somatic cells. While miR-34a-5p inhibits *p62* mRNA expression, REST promotes it.

Autophagy is a key process in maintaining cell homeostasis, and its inhibition often exacerbates pathological processes [68]. In this study, fluoride-induced male reproductive damage was accompanied by autophagy suppression in Sertoli cells. miR-34a-5p inhibition or REST overexpression reversed fluoride-induced autophagy inhibition, highlighting the therapeutical value of targeting miR-34a-5p or REST for mitigating fluoride male reproductive toxicity. Notably, low-concentration fluoride promoted autophagy in Leydig cells, but higher concentrations inhibited it. Autophagy generally plays a protective role in pathological contexts, and its induction often aids in damage repair. For example, hepatocellular carcinoma cells up-regulate TMX2 to induce autophagy and counteract oxidative stress [69]. However, excessive autophagy can lead to autosis, as seen in hypoxic–ischemic neuronal death [70]. In this study, the autophagy promoted by fluoride in Leydig cells is excessive and harmful. The contrasting impacts of fluoride on autophagy in Leydig and Sertoli cells underscore the complexity of autophagy’s role in fluoride-induced toxicity to the male reproductive system.

Admittedly, there are some limitations in the manuscript. The mechanism by which fluoride differences regulate the miR-34a-5p expression in Leydig cells and Sertoli cells also awaits future studies.

## Conclusion

**Fig. 7.**
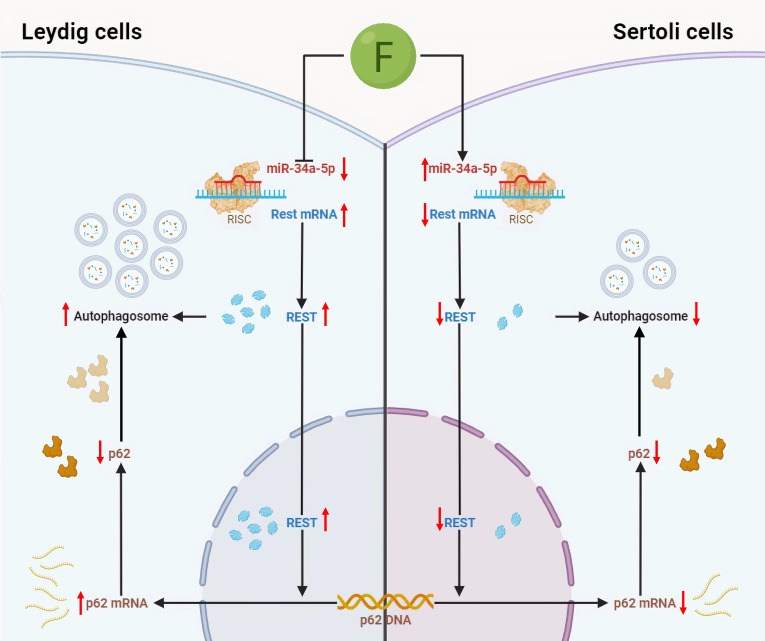
Mechanism diagram of fluoride-induced reproductive injury in male mice by differential regulation of miR-34a-5p targeting REST to modulate autophagy in testicular somatic cells.

Overall, our findings demonstrate that miR-34a-5p controls autophagy in testicular somatic cells by targeting REST. Specifically, fluoride suppresses miR-34a-5p to increase REST and thus promote autophagy in Leydig cells while elevating miR-34a-5p to down-regulate REST and inhibit autophagy in Sertoli cells (Fig. 7). These results provide new insights into the mechanisms of fluoride-induced male reproductive toxicity and identify potential therapeutic targets through the miR-34a-5p-mediated autophagy regulatory pathway.

## Materials and Methods

### Mice and treatments

Twelve 6-week-old male C57BL/6J mice were randomly assigned to either a control group or a NaF group. The control group drank deionized water, while the NaF group received deionized water supplemented with 100 mg/l NaF. All mice were fed water and diet ad libitum in a pathogen-free environment at 25 °C with 50% to 60% humidity. After 18 weeks, the mice were euthanized, and the testicles and epididymis were collected. All animal experiments were conducted following the ethical guidelines approved by the Laboratory Animal Welfare Ethics Review Committee of Shanxi Agricultural University, China (approval no.: IACUC 20200305).

### H&E staining

H&E staining was used to evaluate testicular morphological damage. After paraffin embedding, the fixed testicular tissue was sectioned at a thickness of 5 μm for H&E staining and subsequently observed under an Olympus BX51 microscope.

### Sperm quality assessment

The unilateral epididymis was minced in 1.0 ml of normal saline and incubated for 15 min at 37 °C to make a sperm suspension. The suspension was placed on a slide to make a sperm smear and then fixed with methanol for 5 min. Assessment of sperm quality was conducted following a protocol previously described by our laboratory [71].

### Transmission electron microscopy

The testes were fixed in 1% glutaraldehyde and 4% formaldehyde fixative for 6 h at 4 °C, cut quickly into 1 to 2 mm^3^ cubes, and then fixed for another 6 h. Testicular samples were then processed to meet the TEM requirements described previously [72]. Images of autophagosomes in testicular somatic cells were captured using an HF3300 transmission electron microscope (Hitachi, Tokyo, Japan) at 80 kV.

### Testicular somatic cells and treatments

Murine Leydig cell line (TM3) and murine Sertoli cell line (TM4) were purchased from the American Type Culture Collection (ATCC) (Manassas, USA). The cells were grown in Dulbecco’s modified Eagle’s medium (DMEM)/F-12 containing 2.5% fetal bovine serum (Hyclone, USA), 5% horse serum (Gibco, NZ), and 1% antibiotics (penicillin/streptomycin; Solarbio, China). The cells were grown in a cell incubator maintained at 37 °C, 5% CO_2_, and 95% humidity. Third-generation TM3/TM4 cells were seeded in 6-well plates. At about 80% confluency, the cells were exposed to a complete medium supplemented with 0.125, 0.25, and 0.5 mM NaF and/or 20 μM chloroquine for 24 h and/or pretreated with 20 nM rapamycin for 1 h to construct the model.

### Cell viability assay

Testicular somatic cells were seeded at 6 × 10^4^ cells per well in 96-well culture plates. After 24 h of adhesion, the cells were treated as required. Following the designated exposure period, cell viability was assessed using the cell counting kit-8 (CCK-8) assay.

### Lentivirus infection and modeling

Lentiviruses were purified by Tsingke Biotech. Inhibitor/overexpression-miR-34a-5p, si-REST, and negative control (Table S1) were tagged with enhanced green fluorescent protein (EGFP). OE-REST and its negative control were labeled with PURO. Lentiviruses diluted in a complete medium containing 8 μg/ml polybrene were infected with TM3 [multiplicity of infection (MOI) = 10] and TM4 (MOI = 3) by the 1/2 small-volume infection assay for 1 d. After 2 d, infected cells were screened by fluorescence or puromycin (TM3: 2.0 μg/ml, TM4: 0.5 μg/ml, 3 d).

### Bioinformatics analysis

The NCBI database was used to collect miRNAs associated with fluoride male reproductive damage by searching the keywords “fluoride”, “miRNAs”, and “male reproductive damage”. The GeneCards database (https://www.genecards.org/) was used to obtain miRNAs involved in the process of male reproductive toxicity by searching for keywords “male reproductive toxicity” and “miRNAs”. The HAMDB database (http://hamdb.scbdd.com/) was used to obtain the miRNAs that regulate autophagy. Candidate miRNAs of fluoride affecting autophagy were obtained by intersecting miRNAs from 3 databases for further screening.

The miRWalk version 3 database (http://mirwalk.umm.uni-heidelberg.de/) predicted the potential target genes of miR-34a-5p. The potential transcription factors of p62 were predicted by the ChEA3 database (https://maayanlab.cloud/chea3/). Then, common genes of both were ranked based on the binding coefficient and accessibility, and the top 15 genes were selected to verify the existence of a targeting relationship.

### Fluorescence in situ hybridization

Sections (4 μm) of testicular tissue digested with proteinase K for 15 min were used with FAM-labeled miR-34a-5p or Cy3-labeled *Rest* probes (Servicebio; Table S1). After overnight hybridization at 40 °C, images were viewed and acquired under a biomicroscope (BX51, Olympus, Japan).

### Dual-luciferase reporter gene assay

FOXO1 and REST wild-type and mutant 3′ UTR were biosynthesized by Tsingke Biotech and integrated into pmirGLO plasmid (Thermo Fisher Scientific, USA) to produce the corresponding dual-luciferase reporter vectors. 293T cells were cotransfected with FOXO1 3′ UTR wild-type (WT) or Mut with miR-NC or miR-34a-5p. In addition, REST 3′ UTR WT or Mut was cotransfected with miR-NC or miR-34a-5p into 293T cells. After cotransfection for 48 h, the luciferase activity was detected by a Double-Luciferase Reporter Assay Kit (TransGen).

### Immunofluorescence staining

Testes were sectioned to a thickness of 5 μm, followed by deparaffinization, rehydration, permeabilization, and heat-induced epitope repair. Testicular sections were blocked with 5% bovine serum albumin for 2 h at 25 °C, prior to being incubated overnight with primary antibodies (REST, 1:300, Bioss, China) at 4 °C. Testicular sections were then immersed in Cy3-conjugated secondary antibodies (1:300, Boster, China) for 2 h at room temperature. The nuclei were stained with 4′,6-diamidino-2-phenylindole (DAPI) for 10 min. Subsequently, the testicular sections were examined under a biomicroscope (BX51, Olympus, Japan), and images were captured.

### Immunohistochemistry

Testicular tissue sections after antigen repair were incubated with 3% hydrogen peroxide solution for 15 min and then incubated in 5% normal goat serum for 30 min to block. The sections were incubated with the primary antibodies LC3B (1:300, Proteintech) or p62 (1:300, ABclonal) overnight at 4 °C. Sections were subsequently probed with horseradish peroxidase-conjugated goat anti-rabbit immunoglobulin G (IgG) for 1 h. The 3,3′-diaminobenzidine substrate was used as a chromogenic substrate. After hematoxylin counterstaining, images were collected under a microscope.

### qRT-PCR

Total RNA was isolated from the treated TM3 or TM4 cells by Trizol reagent (Takara, Japan). Total RNA was reverse transcribed into cDNA using EasyScript Uni All-in-One First-Strand cDNA Synthesis SuperMix kit. Special reverse transcription primers and the PrimeScript RT Reagent Kit with gDNA Eraser (Takara, Japan) were used to reverse transcribe miRNAs and *U6*. Next, qRT-PCR was conducted utilizing the PerfectStart Green qPCR SuperMix kit (TransGen, China). Relative mRNA expression was calculated employing the 2^−ΔΔCT^ method and normalized to *U6* or *GAPDH* mRNA levels. The primers employed in this research were synthesized by Tsingke Biotech, China (Table S2).

### WB analysis

Proteins from treated TM3 or TM4 cells were extracted using radioimmunoprecipitation assay (RIPA) lysate containing 1.0 mM phenylmethylsulfonyl fluoride (PMSF) inhibitor (Univ, China). Protein quantification was performed using the bicinchoninic acid (BCA) assay kit (Boster, China). Protein samples (15 μg/lane) were then resolved by sodium dodecyl sulfate–polyacrylamide gel electrophoresis and electrotransferred on nitrocellulose membranes, followed by membrane blocking in 5% skim milk at 25 °C for 2 h, after which the membranes were incubated with the corresponding primary antibodies (REST 1:4,000 Bioss, p62 1:4,000 ABclonal, LC3B 1:2,000 Proteintech) at 4 °C overnight, followed by incubation with goat anti-rabbit IgG (1:6,000 ABclonal). Target stripes in the membranes were examined and visualized on a FluorChem Q system (Alpha Innotech, USA).

### Statistical analysis

GraphPad Prism version 8.0 software was used for statistical analysis. Pairwise comparisons were evaluated using Student’s *t* test, while multi-group differences were evaluated using one-way analysis of variance (ANOVA) followed by Tukey’s multiple comparisons test. All values in the figure are mean ± SEM. Statistical significance was set at *P* < 0.05.

## Data Availability

Data will be made available on request.
