# Supplementary material for: Environmental Fluoride Compromises Male Fertility: Differentially Modulated miR-34a-5p Targets REST to Regulate Autophagy in Testicular Somatic Cells
Source: Research (Wash D C). 2026 Feb 6;9:1113. doi: 10.34133/research.1113 (PMC12877340; doi:10.34133/research.1113)
Supplement: Supplementary 1 — Tables S1 to S5 [file research.1113.f1.docx]

SUPPLEMENTARY MATERIALS

Table S1 The primers sequence

| Name | Sequence (5'→3') |
| --- | --- |
| miR-NC  Top | GATCCTGGCAGTGTCTTAGCTGGTTGTCTTCCTGTCAGAACAACCAGCTAAGACACTGCCATTTTTG |
| miR-NC  Bottom | AATTCAAAAATTCTCCGAACGTGTCACGTTCTGACAGGAAGACGTGACACGTTCGGAGAAG |
| miR-34a-5p  Top | GATCCTGGCAGTGTCTTAGCTGGTTGTCTTCCTGTCAGAACAACCAGCTAAGACACTGCCATTTTTG |
| miR-34a-5p  Bottom | AATTCAAAAATGGCAGTGTCTTAGCTGGTTGTTCTGACAGGAAGACAACCAGCTAAGACACTGCCAG |
| Inhibitor-NC  Top | GATCCtctgaattcttcgatctgcTTTTTGCTTG |
| Inhibitor-NC  Bottom | AATTCAAAAAGCAGATCGAAGAATTCAGAG |
| Inhibitor-miR-34a-5p Top | GATCCACAACCAGCTAAGACACTGCTTTTTG |
| Inhibitor-miR-34a-5p Bottom | AATTCAAAAAGCAGTGTCTTAGCTGGTTGTG |
| si-NC Top | GATCCTGGCAGTGTCTTAGCTGGTTGTCTTCCTGTCAGAACAACCAGCTAAGACACTGCCATTTTTG |
| si-NC Bottom | AATTCAAAAATTCTCCGAACGTGTCACGTTCTGACAGGAAGACGTGACACGTTCGGAGAAG |
| si-REST Top | GATCCGTGTAATCTACAATACCATTTCTTCCTGTCAGAAAATGGTATTGTAGATTACACTTTTTG |
| si-REST Bottom | AATTCAAAAAGTGTAATCTACAATACCATTTTCTGACAGGAAGAAATGGTATTGTAGATTACACG |
| FAM-labeled miR-34a-5p probes | ACAACCAGCTAAGACACTGCCA |
| Cy3-labeled Rest probes | TCGTTGGTTAAGGCCATGCCCATGCCGTCGTGTACGTGCAGATGATGC |

Table S2 The primers sequence

| **Genes** | **Primer sequence (5'→3')** | **Accession No.** | **Product Sizes (bp)** |
| --- | --- | --- | --- |
| *P62* | F: AGGATGGGGACTTGGTTGC | [NM_011018.3](https://www.ncbi.nlm.nih.gov/entrez/viewer.fcgi?db=nucleotide&id=595582148) | 178 |
|  | R: TCACAGATCACATTGGGGTGC |  |  |
| *Rest* | F: CATGGCCTTAACCAACGACAT | NM_006534842 | 134 |
|  | R: CGACCAGGTAATCGCAGCAG |  |  |
| *Foxp1* | F: GGTCTGAGACAAAAAGTAACGGA | NM_001197322 | 72 |
|  | R: CGCACTCTAGTAAGTGGTTGC |  |  |
| *Yy1* | F: CAGTGGTTGAAGAGCAGATCAT | NM_009537 | 77 |
|  | R: AGGGAGTTTCTTGCCTGTCAT |  |  |
| *Smad5* | F: TTGTTCAGAGTAGGAACTGCAAC | NM_001164042 | 114 |
|  | R: GAAGCTGAGCAAACTCCTGAT |  |  |
| *Elf1* | F: TGTCCAACAGAACGACCTAGT | NM_007920 | 151 |
|  | R: CACACAAGCTAGACCAGCATAA |  |  |
| *Klf10* | F: ATGCTCAACTTCGGCGCTT | NM_001289471 | 129 |
|  | R: CGCTTCCACCGCTTCAAAG |  |  |
| *Mef2a* | F: CAGGTGGTGGCAGTCTTGG | NM_001291191 | 132 |
|  | R: TGCTTATCCTTTGGGCATTCAA |  |  |
| *Foxo1* | F: CCCAGGCCGGAGTTTAACC | NM_019739 | 132 |
|  | R: GTTGCTCATAAAGTCGGTGCT |  |  |
| *Creb1* | F: AGCAGCTCATGCAACATCATC | XM_006495650 | 152 |
|  | R: AGTCCTTACAGGAAGACTGAACT |  |  |
| *Terf2* | F: GTGGAACAGCCCTAACGGG | NM_001371065 | 139 |
|  | R: CCACTCGCTTTCTTCTATGGTC |  |  |
| *Nfe2l1* | F: CTGAGTCTGATTGGGGTTCGG | NM_008686 | 111 |
|  | R: GAACTGGGTCTGGGTATAGGC |  |  |
| *Rxra* | F: ATGGACACCAAACATTTCCTGC | NM_011305 | 139 |
|  | R: CCAGTGGAGAGCCGATTCC |  |  |
| *Fos* | F: CGGGTTTCAACGCCGACTA | NM_010234 | 166 |
|  | R: TTGGCACTAGAGACGGACAGA |  |  |
| *Bach1* | F: TGAGTGAGAGTGCGGTATTTGC | NM_007520 | 197 |
|  | R: GTCAGTCTGGCCTACGATTCT |  |  |
| *Foxj2* | F: AGAGAAGACACCCTCCAGATG | NM_021899 | 114 |
|  | R: CCTCGTGAGAGAGTGAGGCTT |  |  |
| *Gapdh* | F: GGTTGTCTCCTGCGACTTCA | [NM_001411843](https://www.ncbi.nlm.nih.gov/entrez/viewer.fcgi?db=nucleotide&id=2295558070) | 183 |
|  | R: TGGTCCAGGGTTTCTTACTCC |  |  |
| *U6* | F: CTCGCTTCGGCAGCACA | [NR_003027.2](https://www.ncbi.nlm.nih.gov/nuccore/NR_003027.2) | 143 |
|  | R: AACGCTTCACGAATTTGCGT |  |  |
| miR-34a-5p | F: CGAGTGGCAGTGTCTTAG | [NR_029751.1](https://www.ncbi.nlm.nih.gov/nuccore/NR_029751.1) | 74 |
|  | R: TATCCAGTGCGTGTCGTG |  |  |
|  | RT:GTCGTATCCAGTGCGTGTCGTGGAGTCGGCAATTGCACTGGATACGACACAACCAG |  |  |
| miR-17-5p | F: CGAGCAAAGTGCTTACAGT | NR_029785.1 | 75 |
|  | R: TATCCAGTGCGTGTCGTG |  |  |
|  | RT:GTCGTATCCAGTGCGTGTCGTGGAGTCGGCAATTGCACTGGATACGACCTACCTGC |  |  |
| miR-130a-3p | F: CCGAGCAGTGCAATGTTAA | NR_029544.1 | 75 |
|  | R: TATCCAGTGCGTGTCGTG |  |  |
|  | RT:GTCGTATCCAGTGCGTGTCGTGGAGTCGGCAATTGCACTGGATACGACATGCCCTT |  |  |
| miR-423-5p | F: GAGTGAGGGGCAGAGAGC | NR_030756.1 | 74 |
|  | R: TATCCAGTGCGTGTCGTG |  |  |
|  | RT:GTCGTATCCAGTGCGTGTCGTGGAGTCGGCAATTGCACTGGATACGACAAAGTCTC |  |  |
| miR-484 | F: CGAGTCAGGCTCAGTCCC | NR_030252.1 | 74 |
|  | R: TATCCAGTGCGTGTCGTG |  |  |
|  | RT:GTCGTATCCAGTGCGTGTCGTGGAGTCGGCAATTGCACTGGATACGACATCGGGAG |  |  |
| miR-204-5p | F: CCGAGTTCCCTTTGTCATC | NR_029591.1 | 75 |
|  | R: TATCCAGTGCGTGTCGTG |  |  |
|  | RT:GTCGTATCCAGTGCGTGTCGTGGAGTCGGCAATTGCACTGGATACGACAGGCATAG |  |  |
| miR-125b-5p | F: CCGAGTCCCTGAGACCCTA | [NR_107881.1](https://www.ncbi.nlm.nih.gov/nuccore/NR_107881.1) | 75 |
|  | R: TATCCAGTGCGTGTCGTG |  |  |
|  | RT:GTCGTATCCAGTGCGTGTCGTGGAGTCGGCAATTGCACTGGATACGACTCACAAGT |  |  |
| miR-140-3p | F: GCCGAGTACCACAGGGTAG | [NR_029553.1](https://www.ncbi.nlm.nih.gov/nuccore/NR_029553.1) | 75 |
|  | R: TATCCAGTGCGTGTCGTG |  |  |
|  | RT:GTCGTATCCAGTGCGTGTCGTGGAGTCGGCAATTGCACTGGATACGACCCGTGGTT |  |  |
| miR-199a-5p | F: CGAGCCCAGTGTTCAGACT | NR_107954.1 | 75 |
|  | R: TATCCAGTGCGTGTCGTG |  |  |
|  | RT:GTCGTATCCAGTGCGTGTCGTGGAGTCGGCAATTGCACTGGATACGACGAACAGGT |  |  |
| miR-142a-3p | F: CCGAGTGTAGTGTTTCCTAC | [NR_029555.1](https://www.ncbi.nlm.nih.gov/nuccore/NR_029555.1) | 76 |
|  | R: TATCCAGTGCGTGTCGTG |  |  |
|  | RT:GTCGTATCCAGTGCGTGTCGTGGAGTCGGCAATTGCACTGGATACGACTCCATAAA |  |  |

**F**, forward primers; **R**, reverse primers; **RT**, reverse transcription primers.

Table S3 miRNAs related to fluoride-induced male reproductive injury

| **Gene Symbol** | **GC Id** | **Relevance score** |
| --- | --- | --- |
| miR-34a-5p | GC01M009151 | 26.98823929 |
| miR-17-5p | GC13P091350 | 23.93447495 |
| miR-140-3p | GC16P114132 | 19.8539505 |
| miR-142a-3p | GC17M058331 | 18.87212944 |
| miR-451a | GC17M028861 | 17.29108429 |
| miR-204-5p | GC09M070809 | 13.86263847 |
| miR-423-5p | GC17P030117 | 12.34772873 |
| miR-130a-3p | GC11P057641 | 11.81768608 |
| miR-484 | GC16P112597 | 2.088090897 |

Table S4 Autophagy-related candidate miRNAs

| Gene  Symbol | Relevance  score | Autophagy  regulation | Reference |
| --- | --- | --- | --- |
| miR-34a-5p | 26.98823929 | Yes | morgado al, xavier jm, dionísio pa, ribeiro mf, dias rb, sebastião am, solá s, rodrigues cm****microrna-34a modulates neural stem cell differentiation by regulating expression of synaptic and autophagic proteins.****mol neurobiol.2015;51(3):1168-83. doi: 10.1007/s12035-014-8794-6. ****24973144 |
| miR-17-5p | 23.93447495 | Yes | Du WW, Yang W, Fang L, Xuan J, Li H****miR-17 extends mouse lifespan by inhibiting senescence signaling mediated by MKP7.****Cell Death Dis. 2014 Jul 31;5:e1355. doi: 10.1038/cddis.2014.305.****25077541 |
| miR-140-3p | 19.8539505 | Yes | lin sh, song w, cressatti m, zukor h, wang e, schipper hm****heme oxygenase-1 modulates microrna expression in cultured astroglia: implications for chronic brain disorders.****glia.2015 Jul;63(7):1270-84. doi: 10.1002/glia.22823. ****25820186 |
| miR-142a-3p | 18.87212944 | Yes | zhai z, wu f, dong f, chuang ay, messer js, boone dl, kwon jh****human autophagy gene atg16l1 is post-transcriptionally regulated by mir142-3p.****autophagy.2014 Mar;10(3):468-79. doi: 10.4161/auto.27553. ****24401604 |
| miR-204-5p | 13.86263847 | Yes | Sümbül AT, Göğebakan B, Ergün S, Yengil E, Batmacı CY, Tonyalı Ö, Yaldız M.****miR-204-5p expression in colorectal cancer: an autophagy-associated gene.****Tumour Biol. 2014 Dec;35(12):12713-9. doi: 10.1007/s13277-014-2596-3.****25209181 |
| miR-423-5p | 12.34772873 | Yes | stiuso p, potenza n, lombardi a, ferrandino i, monaco a, zappavigna s, vanacore d, mosca n, castiello f, porto s, addeo r, prete sd, de vita f, russo a, caraglia m****microrna-423-5p promotes autophagy in cancer cells and is increased in serum from hepatocarcinoma patients treated with sorafenib.****mol ther nucleic acids.2015 Mar 17;4:e233. doi: 10.1038/mtna.2015.8.****25782064 |
| miR-130a-3p | 11.81768608 | Yes | kovaleva v, mora r, park yj, plass c, chiramel ai, bartenschlager r, Döhner H, stilgenbauer s, pscherer a, lichter p, seiffert m****mirna-130a targets atg2b and dicer1 to inhibit autophagy and trigger killing of chronic lymphocytic leukemia cells.****cancer res.2012 Apr 1;72(7):1763-72. doi: 10.1158/0008-5472.CAN-11-3671. ****22350415 |
| miR-484 | 2.088090897 | Yes | helwak a, kudla g, dudnakova t, tollervey d****mapping the human mirna interactome by clash reveals frequent noncanonical binding.****cell.2013 Apr 25;153(3):654-65. doi: 10.1016/j.cell.2013.03.043.****23622248 |
| miR-451a | 17.29108429 | No | / |

Table S5 Potential target genes of miR-34a-5p

| Gene symbol | Refseq id | Binding coefficient | Accessibility |
| --- | --- | --- | --- |
| *Foxo1* | NM_019739 | 1 | 0.03077867 |
| *Elf1* | NM_007920 | 1 | 0.009975595 |
| *Rxra* | NM_011305 | 1 | 0.008513623 |
| *Rest* | XM_006534842 | 1 | 0.002418825 |
| *Klf10* | NM_001289471 | 1 | 0.001811844 |
| *Bach1* | NM_007520 | 1 | 0.001079065 |
| *Foxp1* | NM_001197322 | 1 | 0.000240803 |
| *Yy1* | NM_009537 | 1 | 0.00021078 |
| *Creb1* | XM_006495650 | 1 | 0.00015761 |
| *Mef2A* | NM_001291191 | 1 | 0.000137898 |
| *Foxj2* | NM_021899 | 1 | 0.0000908 |
| *Nfe2L1* | NM_008686 | 1 | 0.0000773 |
| *Fos* | NM_010234 | 1 | 0.0000676 |
| *Smad5* | NM_001164042 | 1 | 0.000039 |
| *Terf2* | NM_001371065 | 1 | 0.00000692 |
| *Stat2* | NM_019963 | 1 | 0.00000108 |
| *Tsc22D1* | NM_009366 | 1 | 7.04E-08 |
| *Mitf* | NM_008601 | 0.923076923 | 0.02965819 |
| *Mxi1* | NM_001360345 | 0.923076923 | 0.01103547 |
| *Bhlhe40* | NM_011498 | 0.923076923 | 0.005269939 |
| *Zfx* | NM_001044386 | 0.923076923 | 0.00457289 |
| *Foxp2* | NM_053242 | 0.923076923 | 0.001438026 |
| *Sox6* | NM_011445 | 0.923076923 | 0.000806014 |
| *Zbtb43* | NM_001025594 | 0.923076923 | 0.000489275 |
| *Irf1* | NM_001159393 | 0.923076923 | 0.0000556 |
| *Ppard* | XM_036160411 | 0.923076923 | 0.000000265 |
| *Tbp* | NM_013684 | 0.923076923 | 0.0000002 |
| *Usf2* | NM_011680 | 0.884615385 | 0.000241254 |
| *Gabpa* | NM_008065 | 0.884615385 | 0.0000322 |
| *Pknox1* | XM_006523854 | 0.846153846 | 0.01787135 |
| *Tal1* | XM_006502912 | 0.846153846 | 0.004715375 |
| *Rxrb* | NM_001205215 | 0.846153846 | 0.004421713 |
| *Etv1* | NM_007960 | 0.846153846 | 0.004066162 |
| *Max* | XM_006515523 | 0.846153846 | 0.000279903 |
| *Mnt* | NM_010813 | 0.846153846 | 0.000145144 |
| *Bcl11A* | NM_016707 | 0.846153846 | 0.000139311 |
| *Pbx3* | NM_016768 | 0.846153846 | 0.000130947 |
| *Pou2F1* | NM_011137 | 0.846153846 | 0.0000629 |
| *Rfx5* | NM_017395 | 0.846153846 | 0.0000472 |
| *Cebpd* | NM_007679 | 0.846153846 | 0.0000434 |
| *Tcf12* | NM_011544 | 0.846153846 | 0.0000211 |
| *Tcf7L2* | XM_017318114 | 0.846153846 | 0.0000115 |
| *Nr3C1* | NM_001361209 | 0.846153846 | 0.0000111 |
| *Tfap4* | NM_031182 | 0.846153846 | 0.00000876 |
| *Crem* | XM_006525579 | 0.846153846 | 0.00000494 |
| *Nrf1* | NM_001164228 | 0.846153846 | 0.00000158 |
| *Ets1* | NM_001372534 | 0.846153846 | 0.00000112 |
| *Myc* | NM_010849 | 0.846153846 | 0.000000919 |
